# Prescription Department

**Published:** 1894-04

**Authors:** 


GRANULAR CONJUNCTIVITIS.

℞ Hydrag. oxid. flav., gr. iij.

Zinci. oxidi.,

Thymol.,

Cocain. hydrochlorat., āā gr. iss.

Camphoræ, gr. ss.

Vaselini, ʒ vj.

M. Sig. Apply locally.—*La Riforma Med.*

EPISTAXIS.

The method of Dr. Rougier is to paint the spot from which the hemorrhage seems to come with:

℞ Collodion, ℥ iij.
Acid. corbolic,
Acid. benzoic,
Acid. tannic, āā gr. lxxv.—M.

This preparation coagulates albumen instantaneously, and its use is not painful. The author also employs it after removal of adenoid tumors, tonsillotomy, etc.—*La Méd. Mod.-Ex.*

DIABETES INSIPIDUS.

℞ Sodii salicylic., ℥ ss.

Tinct. opii, ℈ j.

Aq. menth. pip., f ℥ v.

Glycerin., f ℥ j.

M. Sig. Teaspoonful four times a day.—*Medical Review.*

NIGHT-SWEATS OF PHTHISIS.

M. Alb. Robin prescribes:

℞ Pulv. agarici, gr. viij.

Zinc. oxid., gr. iss.

Pulv. camphor., gr. ⅓.

M. Sig. For one cachet. To be taken on going to bed.—*La Médecine Moderne.—Ex.*

MENTAL EXCITEMENT OF HYSTERIA.

Dr. Blocq prescribes:
℞ Camphor. monobrom, gr. xlv.
Extr. quassiæ, ʒ ss.
Syrup belladonn., q. s.
M. et ft. pil. no. xxx.
Sig. One, two or three pills a day.—*La Riforma Medina.—Ex.*

OXALIC ACID AS AN EMMENAGOGUE.

By G. A. F.

℞ Acidi oxalici, grms. 2.
Aquæ dest., grms. 400.
Glycerini, grms. 40.
Syr. simplicis, grms. 60.

M. Sig. Two to four tablespoonfuls every hour.—*Ex.*

PROPHYLACTIC AGAINST FREQUENTLY RECURRING TONSILLITIS.

℞ Acidi carbolici (cryst.), grms. 5.

Alcoholis, grms. 10.

Ol. menth. pip., gtt. j.

M. Sig. Ten drops in a cup of warm water as a gargle, morning and evening.—*Ex.*

NASAL CATARRH.

By S. D. Donaho, Collinsville, Tenn:

℞ Eucalyptol (Sanders & Sons), gtt. xv.
Aquæ, ℥ j.
Alcoholis, q. s.

M. Sig. Use as spray three or four times daily.—*Ex.*

DYSENTERY IN CHILDREN.

Dr. S. S. Adams, of Washington, D. C., says in Food: If the pain and straining are intense, relief may be derived from the following:

℞ Cocain. muriat., gr. ij.
Extract. ergot. aq., gr. x.
Extract. opii aq., gr. ij.
Aristol., gr. v.
Olei theobrom., q. s.

M. Ft. suppos. no. x.

Sig. One every two or three hours.—*Ex.*

INSOMNIA OF CHILDREN.

℞ Chloralis, gr. ij.
Tr. moschi.,
Tr. valerianæ, āā gtt. xx.
Aquæ dest., ℥ j.

M. Sig. Inject the entire quantity into the rectum, and if necessary, the dose may be repeated if sleep does not ensue in the course of two or three hours.—*Ex.*

